# Supplementary material for: The spectral features of EEG responses to transcranial magnetic stimulation of the primary motor cortex depend on the amplitude of the motor evoked potentials
Source: PLoS One. 2017 Sep 14;12(9):e0184910. doi: 10.1371/journal.pone.0184910 (PMC5599017; doi:10.1371/journal.pone.0184910)
Supplement: S1 Text — (PDF) [file pone.0184910.s001.pdf]

Additional results

TMS over the medial M1 (leg area) leads to and ERD only in the presence of muscle twitch

Resting Motor Threshold (RMT) is defined as the minimum stimulus intensity that produces a minimal motor evoked response (about 50 µV in at least 5 out of 10 trials) while the target test muscle is at rest [1]. RMT changes depending on the considered target muscle due to scalp-to-cortex distance [2] and higher stimulation intensity is needed to induce a muscle twitch when perturbing the medial portion of the M1 (e.g. the leg motor area). Along the same line, here we show that, when stimulating medial motor cortex, TMS-induced ERD can be obtained just stimulating at the RMT of the targeted muscle (here the right quadriceps). To do this, in one representative subject, once obtained RMT by stimulating APB area (RMT_{APB}), we moved to a more medial area, performing a first TMS/EEG recording by targeting the portion of M1 corresponding to right quadriceps. Then, we increased the stimulation intensity until reaching a selective MEP of the right quadriceps ($RMT_{Quadriceps}$) muscle without involving APB activation.

[1] Rossini PM, Burke D, Chen R, Cohen LG, Daskalakis Z, Di Iorio R, et al. Non-invasive electrical and magnetic stimulation of the brain, spinal cord, roots and peripheral nerves: Basic principles and procedures for routine clinical and research application. An updated report from an I.F.C.N. Committee. Clin Neurophysiol 2015;126:1071–107. doi:10.1016/j.clinph.2015.02.001.

[2] Herbsman T, Forster L, Molnar C, Dougherty R, Christie D, Koola J, et al. Motor Threshold in Transcranial Magnetic Stimulation: The Impact of White Matter Fiber Orientation and Skull-to-Cortex Distance. Hum Brain Mapp 2009;30:2044–55. doi:10.1002/hbm.20649.
