# Supplementary figures and images for: The spectral features of EEG responses to transcranial magnetic stimulation of the primary motor cortex depend on the amplitude of the motor evoked potentials
Source: PLoS One. 2017 Sep 14;12(9):e0184910. doi: 10.1371/journal.pone.0184910 (PMC5599017; doi:10.1371/journal.pone.0184910)

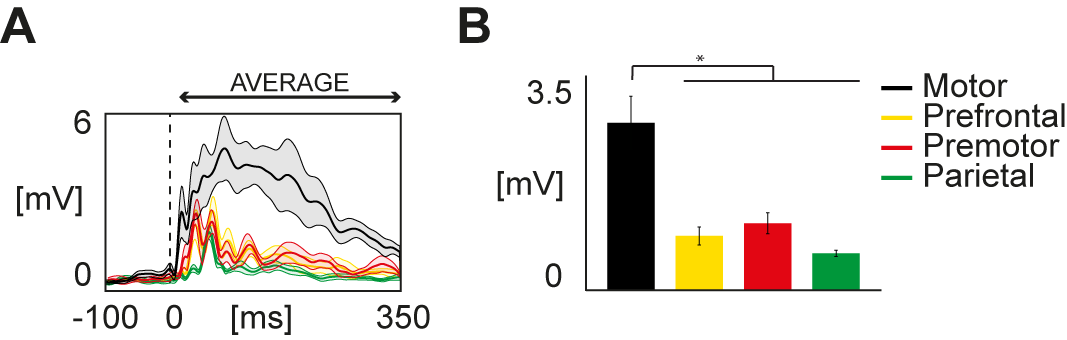

Supplement: S1 Fig — (A) Grand-average of GMFP for each stimulated area. Thick traces indicate the grand-average GMFP across subjects (±SE, color-coded shaded regions). Responses recorded after the stimulation of different cortical areas are color coded as follows: motor in black, prefrontal in yellow, premotor in red, parietal in green. (B) For each stimulated area, the GMFP values averaged between 8 and 350 ms post-TMS are shown in the bar histogram (mean ± SE). Asterisks indicate statistically significant differences (* p<0.05, Wilcoxon signed rank test). Bars are color coded as in Panel A. (TIF) [file pone.0184910.s002.tif]

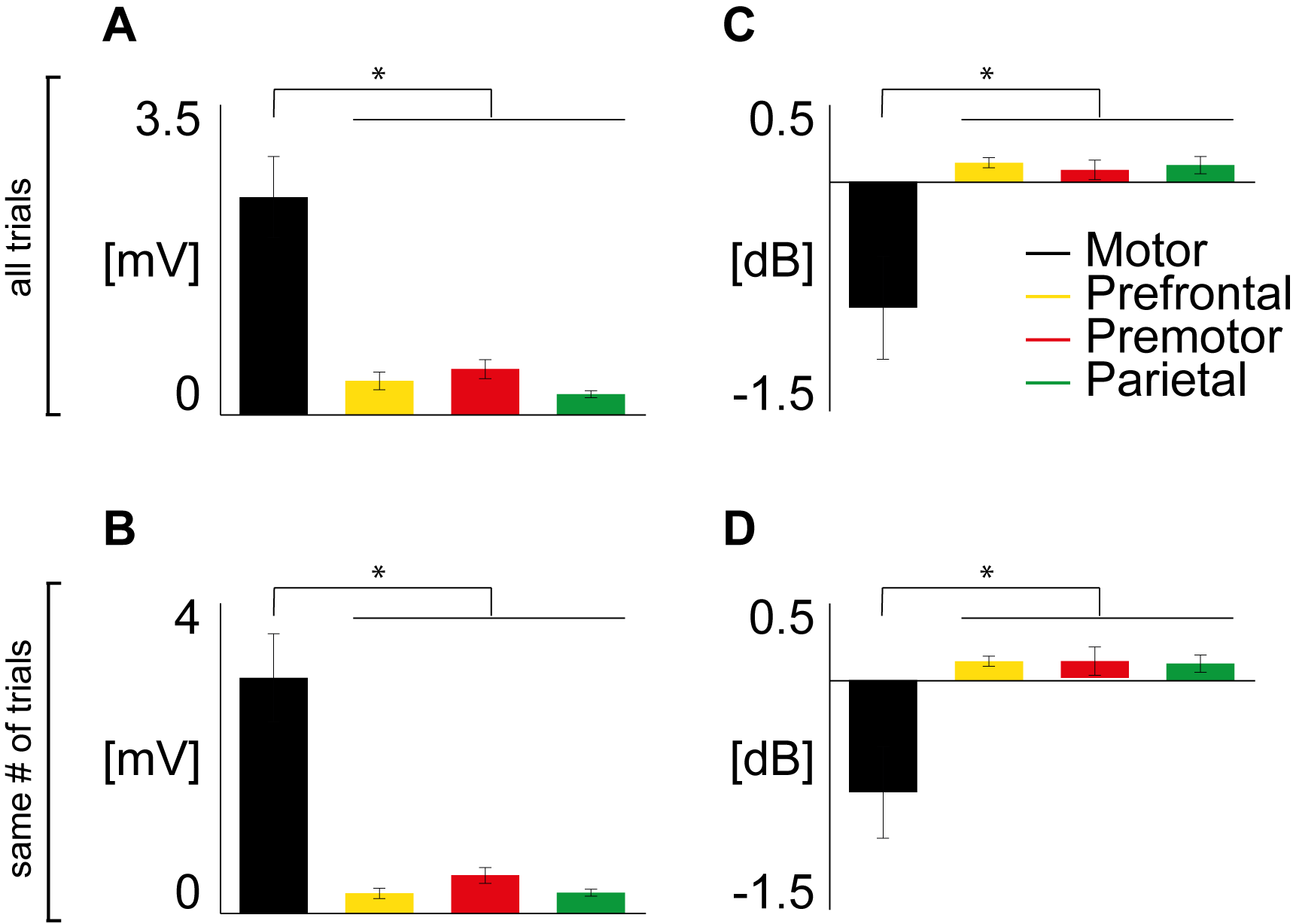

Supplement: S2 Fig — Asterisks indicate statistically significant differences (* p<0.05, Wilcoxon signed rank test). Bars are color coded as in Fig 1. Using the same color coding for each stimulated area, the grand-average (±SE) of the averaged ERD, calculated using all artifact free trials (panel C) and the same number of trials across stimulation site (panel D). Asterisks indicate statistically significant differences (* p<0.05, Wilcoxon signed rank test). (TIF) [file pone.0184910.s003.tif]

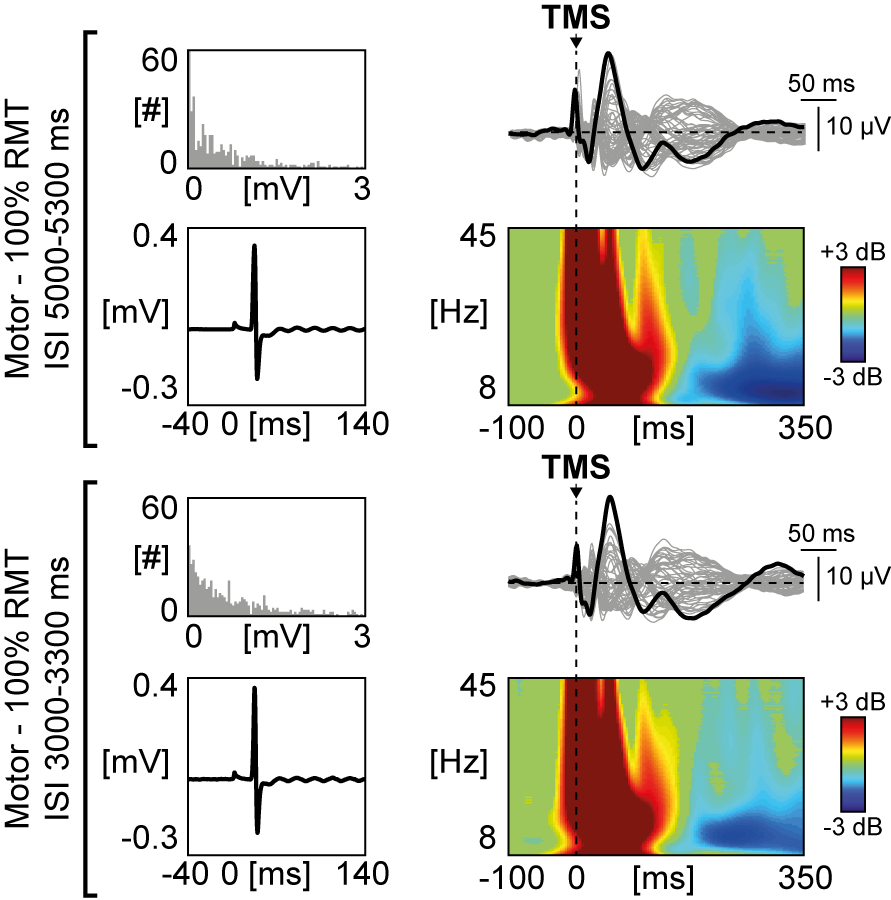

Supplement: S3 Fig — For both ISIs, the distribution of peak-to-peak MEP amplitude of all artifact free trials (left top) and the corresponding average MEP (left bottom), the butterfly plots of all channels (right top, grey traces), the TEPs recorded at the channel closest to the stimulation site (right top, black traces) and the corresponding ERSPs (right bottom) are shown. Wavelet Transform (Morlet, 3.5 cycles) was applied at the single trial level. Significance threshold for bootstrap statistics is set at α < 0.01. Non-significant activity was set to zero (green), red colors indicate a significant increase with respect to the baseline, while blue colors indicate a significant reduction with respect to the baseline. The dashed vertical line indicates the timing of the TMS pulse. (TIF) [file pone.0184910.s004.tif]

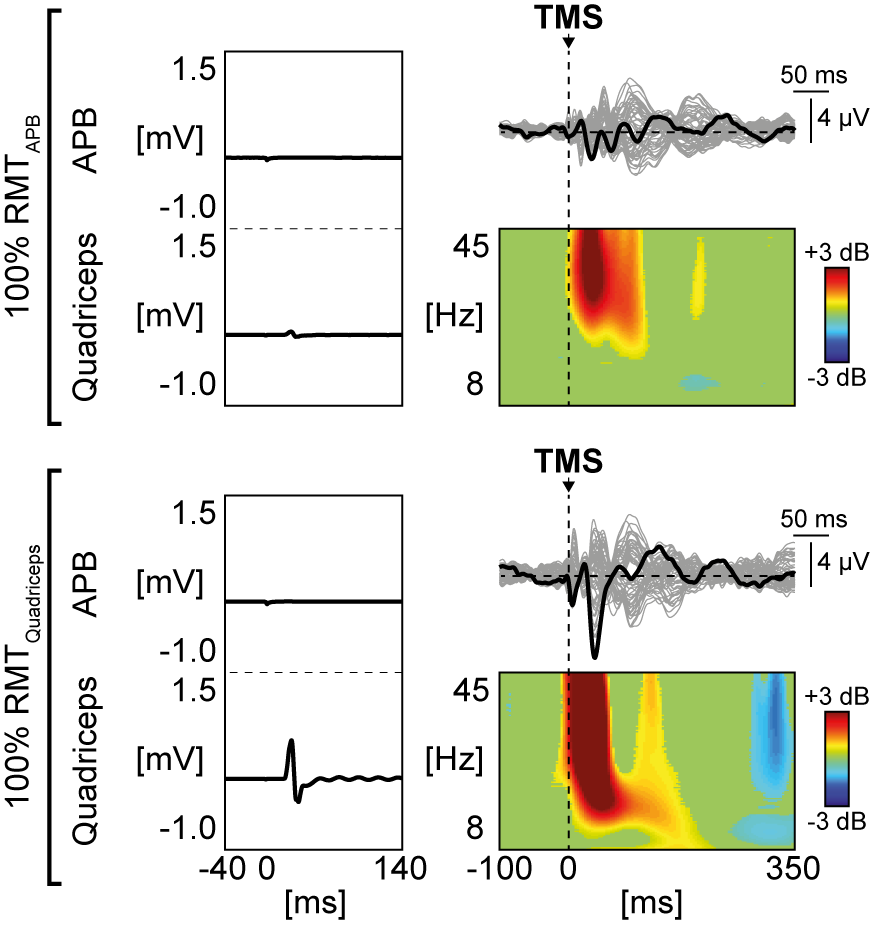

Supplement: S4 Fig — (TIF) [file pone.0184910.s005.tif]
